# 二代测序技术协助诊断急性白血病合并慢性播散性念珠菌病3例

**DOI:** 10.3760/cma.j.issn.0253-2727.2022.06.014

**Published:** 2022-06

**Authors:** 建萍 罗, 小晶 林, 杨 沈

**Affiliations:** 上海交通大学医学院附属瑞金医院血液科，上海 200025 Department of Hematology, Ruijin Hospital, Shanghai Jiao Tong University School of Medicine, Shanghai 200025, China

慢性播散性念珠菌病（CDC）是侵袭性真菌病（IFD）的一种特殊临床和病理表现类型，靶器官主要为肝、脾等深部组织，偶累及肾脏，既往又称为肝脾念珠菌病（HSC）[1]。CDC在临床上缺乏特异性症状和体征，病原学诊断率低，确诊困难且伴有较高的病死率。二代测序技术（NGS）因高通量、高灵敏度、低成本等优势，目前广泛应用于感染性疾病病原体的检测，但在国内应用NGS诊断血液肿瘤合并CDC的报道较少。现对我科收治的3例经NGS诊断的急性白血病（AL）合并CDC患者进行分析并复习相关文献如下。

## 病例资料

例1，女，16岁，因“头晕伴乏力”至当地医院查血常规提示WBC 9.86×10^9^/L，HGB 36 g/L，PLT 21×10^9^/L，骨髓穿刺示原始+幼稚淋巴细胞占0.725，血涂片中原始+幼稚淋巴细胞占0.670，余临床特征见表1。于2021年2月10日予VILP方案（长春地辛4 mg/d，d 1；伊达比星12 mg/d，d 1～3；培门冬酶3 000 U/d，d 7；地塞米松15 mg/d、d 1～14，10 mg/d、d 15～19，7.5 mg/d、d 20～22，5 mg/d、d 23～25）化疗，化疗后粒细胞缺乏伴发热、肛周不适，先后予头孢哌酮钠舒巴坦钠、亚胺培南西司他丁钠抗感染治疗。诱导化疗第14天复查骨髓涂片示原始+幼稚淋巴细胞占0.260，流式细胞术提示异常细胞占8.71％，诱导化疗第23天，复查WBC 2.1×10^9^/L，HGB 70 g/L，PLT 91×10^9^/L，患者无发热，予出院。出院后反复发热，热峰38.5 °C，无咳嗽、咳痰，胸闷、腹痛，3月12日再次入院。血常规示WBC 1.56×10^9^/L，ANC 1.07×10^9^/L，HGB 52 g/L，PLT 531×10^9^/L，C反应蛋白（CRP）56.1 mg/L；骨髓象示骨髓增生减低，流式细胞术微小残留病（MRD）0.47％。给予亚胺培南西司他丁钠+万古霉素+卡泊芬净抗感染治疗，体温未控制。胸部CT提示两肺斑片模糊影，炎症改变可能。上腹部CT提示肝脏多发小低密度影，脾脏稍大。考虑肝脏感染性病变，再次行上腹部MR平扫+增强提示肝脾肿大，肝脏、脾脏多发结节灶。期间体温峰值高于39.0 °C。3月24日行CT引导下肝穿刺活检，提示肝组织局灶坏死，纤维组织增生伴较多中性粒细胞及嗜酸性粒细胞浸润，未见肯定的恶性成分；组织涂片未检出细菌、真菌、抗酸杆菌，组织培养2 d未观察到细菌、真菌生长；肝穿刺组织NGS检出白色念珠菌（序列数2），外周血二代测序及血培养均阴性。后于外院接受氟胞嘧啶+卡泊芬净抗念珠菌治疗，1周后体温峰值下降至38.2 °C，继续联合抗念珠菌治疗2周，体温峰值降至37.3 °C，后体温平稳。抗念珠菌治疗3周，复查肝脏MR提示局部高信号病灶较前增多增大，但患者临床症状好转，WBC恢复正常，考虑为免疫重建炎症反应综合征。继续抗念珠菌治疗同时行Hyper-CVAD方案巩固化疗，期间未出现严重感染。抗念珠菌治疗2个月复查肝脏MR，提示局部病灶较前有所吸收。

例2，女，39岁，2021年1月18日因“乏力伴齿龈出血及皮肤瘀斑”至当地医院查血常规提示WBC 0.98×10^9^/L，RBC 1.23×10^12^/L，HGB 43 g/L，PLT 11×10^9^/L，骨髓象示异常早幼粒细胞占0.675，血涂片中异常早幼粒细胞占0.51，提示急性早幼粒细胞白血病，余临床特征见表1。2021年1月21日开始给予全反式维甲酸（ATRA）+三氧化二砷（ATO）双诱导治疗，期间体重增加，反复高热，考虑分化综合征，予地塞米松、加强利尿对症治疗，辅以哌拉西林钠他唑巴坦钠、替加环素、伏立康唑抗感染治疗后好转出院，出院后继续口服ATRA，WBC正常后停用。出院1周后再次发热，体温峰值达40 °C，3月5日骨髓象示APL趋向缓解。3月6日血常规示WBC 9.93×10^9^/L，中性粒细胞比值85.9％，HGB 83 g/L，PLT 183×10^9^/L，CRP 78 mg/L。胸部CT未见明显异常，腹部CT示肝内多发低密度影，脾脏内稍低密度影。考虑肝脾感染性病变，给予亚胺培南西司他丁钠+替考拉宁+卡泊芬净抗感染治疗，体温无改善，多次血培养均阴性，拟行肝穿刺，但因病灶较小，穿刺难度大，故行外周血NGS，检出近平滑念珠菌（序列数3）。继续给予亚胺培南西司他丁钠+替考拉宁+卡泊芬净治疗，体温控制不佳，更换为两性霉素B脂质体+米卡芬净+亚胺培南西司他丁钠+万古霉素，仍高热（39.0 °C以上），后转至外院予两性霉素B脂质体+卡泊芬净抗真菌治疗，约2周体温下降至38.0 °C，出院后回当地医院继续治疗，失访。

**表1:** 3例急性白血病合并播散性念珠菌病患者临床特征

例号	诊断	LAIP特征	基因改变	染色体核型	化疗方案	诱导化疗后疾病状态
1	ALL	CD9^st+^CD38^+^CD34^−^CD10^p+^CD20^−^CD19^+^CD45^dim^	MEF2D-BCL9、NRAS（20.4%）、ARID5B（64.1%）、ZRSR2（55.4%）	46,XX[10]	VILP	缓解
2	APL	无	PML/RARa、FLT-ITD、ASXL1、TET2、TP53、WT1	46,XX,t(15;17)(q24;q21)[10]/46,XX[1]	ATRA+ATO	缓解
3	AML	CD7^+^CD34p^+^CD117^+^CD13^+^CD33^+^HLA-DR^+^CD45^dim^	CEBPA-C端（50.9%）、CEBPA-N端（50.3%）、WT1（5%）	46,XY[20]	IA	缓解

注：ALL：急性淋巴细胞白血病；APL：急性早幼粒细胞白血病；AML：急性髓系白血病（非APL）；LAIP：白血病相关免疫表型；st+：抗原表达强度明显强于正常对照标本；p+：抗原表达率在20％～80％之间；dim：抗原表达强度明显弱于正常对照标本；VILP：长春地辛+伊达比星+培门冬酶+地塞米松；ATRA：全反式维甲酸；ATO：三氧化二砷；IA：伊达比星+阿糖胞苷

例3，男，45岁，因“皮肤瘀斑、瘀点2个月”至我院就诊，血常规示WBC 75.6×10^9^/L，HGB 94 g/L，PLT 12×10^9^/L，骨髓象示原始细胞占0.750，外周血涂片示原始细胞占0.95，余临床特征见表1。于2021年3月3日开始IA方案（伊达比星 15 mg/d、d 1，20 mg/d、d 2～3；阿糖胞苷190 mg/d，d 1～7）化疗，化疗第4天出现发热，体温39.3 °C，给予头孢哌酮钠舒巴坦钠+阿米卡星抗感染治疗，3月12日仍发热，体温39.0 °C，血培养及导管血培养见蜡样芽孢杆菌，给予亚胺培南西司他丁钠+利奈唑胺抗感染治疗并拔除PICC导管，2天后仍有发热，加用伏立康唑，体温控制后出院。出院后反复发热，体温峰值达40 °C，伴畏寒、寒战。4月10日再次入院，骨髓象示完全缓解，流式细胞术MRD<0.01％，基因提示CEBPA双突变阴性。胸部CT示两肺散在少许条片、结节及条索，双侧胸膜局限性增厚，右侧少量胸腔积液。上腹部CT提示对比原片，肝多发低密度影，胆囊炎，脾脏密度不均，左肾低密度影，右肾高密度影。多次血培养阴性。4月12日行CT引导下肝穿刺活检，提示送检为炎性渗出、坏死及肉芽组织，未见肯定的异型成分；组织涂片未检出细菌、真菌、抗酸杆菌，组织培养2 d未见细菌、真菌生长；肝穿刺组织NGS检出白色念珠菌（序列数77），给予伏立康唑联合米卡芬净抗念珠菌治疗，3 d后体温峰值由39.6 °C降至38.6 °C，发热间歇期延长至72 h，后予伏立康唑联合卡泊芬净继续抗念珠菌治疗，1周左右体温下降至37.5 °C，后体温平稳，未再出现发热。抗念珠菌治疗2周复查上腹部增强CT提示肝脾多发异常强化密度灶。抗念珠菌治疗6周复查上腹部增强CT提示肝脾多发异常强化密度灶较前缩小。后期按疗程巩固化疗，化疗期间维持原方案抗念珠菌治疗，体温平稳，出院后口服伏立康唑片，白血病病情稳定。

## 讨论及文献复习

CDC的致病菌属以白色念珠菌最多见，占50％以上，其次是热带念珠菌和光滑念珠菌[2]。血液肿瘤患者由于强烈诱导化疗引起粒细胞缺乏及胃肠道黏膜受损，念珠菌经受损黏膜进入血液，随血流播散至肝、脾等器官[3]。据国内外文献报道，AL化疗后CDC的发生率为3％～29％[4]–[5]。近年来，随着化疗强度增大和造血干细胞移植的发展，CDC的发生率有升高趋势。病原学证据如血培养或肝、脾穿刺组织培养及组织病理学是CDC诊断的金标准，但急性念珠菌血症期血培养阳性率约为32％，CDC期的阳性率仅有20％左右[6]，可能与血液肿瘤患者大多在诱导治疗期间预防性或经验性使用抗真菌药物有关。文献报道CDC与免疫重建炎症反应综合征有关，糖皮质激素能修复免疫性损伤，改善临床症状及疾病进程[7]–[8]。当患者粒细胞缺乏恢复，仍持续发热应考虑该病可能，影像学检查可发现肝、脾甚至双肾多发感染灶。组织穿刺活检提示病变中心为坏死物，周围有中性粒细胞浸润，过碘酸希夫、六胺银及HE染色在坏死中心发现念珠菌芽孢或菌丝可确诊。但穿刺取材不一定处于病灶结节中央，导致确诊率降低，临床应用有一定的局限性。

本研究3例AL患者，诱导化疗后处于粒细胞缺乏恢复期，反复高热，多次血清G/GM试验、血培养均阴性，上腹部CT/MR增强提示肝脾多发结节灶。1例因病灶较小未行肝穿刺，但外周血NGS检出近平滑念珠菌序列，2例行CT引导下肝穿刺活检，组织病理及真菌培养未见念珠菌芽孢或菌丝，但病灶中有坏死物，周围有中性粒细胞浸润，与文献报道一致[8]，NGS均检出白色念珠菌序列。例2的后续治疗资料失访。例1和例3经抗真菌治疗临床症状好转、影像学改善，进一步证实了播散性念珠菌病的诊断。影像学方面，增强MR对于病灶的发现具有更加清晰的特点。病灶在T1WI多呈低信号，T2WI呈等信号或高信号。肝脏真菌感染MR上较为特征性的表现为病灶周围环形强化，由小脓肿外周被淋巴、巨噬细胞或巨细胞包裹并纤维化形成[9]–[10]。

胡必杰团队开展的一项关于mNGS应用于感染性疾病诊治的大规模研究发现，mNGS在感染性疾病尤其在结核分枝杆菌、病毒、厌氧菌和真菌的检测敏感度和特异度可达到50.7％和85. 7％，受抗菌药物使用的影响小于传统培养[11]。另一项研究显示，肺组织二代测序对细菌感染的灵敏度可达100％，特异度为76.5％；对真菌感染的灵敏度为57.1％，特异度为61.5％；对细菌和真菌的阳性预测值分别为42.9％和44.4％，阴性预测值分别为100％和72.7％[12]。程军等[13]研究不同方法诊断31例感染性心内膜炎（IE）的诊断效能，与传统培养方法相比，NGS检测IE瓣膜赘生物的灵敏度更高，且时间短。

然而，NGS作为一种新的病原学诊断技术也存在一些问题：①宿主核酸干扰：所有临床样本均包含大量人类基因组序列，消除宿主背景核酸干扰可进一步提高病原体检测灵敏度并降低检测成本。Hasan等[14]发现用0.025％的非离子表面活性剂皂甙处理的样本大大减少了人类基因组读数，使微生物比率增加20～650倍。② 核酸提取方法：真菌及细胞内微生物如分枝杆菌属细胞壁较厚，在核酸提取过程中破壁困难核酸序列检出率低。黄洁等[15]指出对真菌及细胞内微生物提取DNA的过程可能影响RNA的检出率，需要进一步优化核酸提取试剂盒。③质量控制及生物信息分析：NGS是复杂的多步骤测试，需要多个质量控制指标，包括样品、外部、内部、污染对照及文库质量、测序质量指标。生物信息分析流程必须经过性能确认和验证，包括将对照和患者数据集进行分析比较，通过常规临床测试确定最终结果的准确性[16]，目前尚缺乏标准。④数据解读：NGS结果解读规范，包括序列数阈值，灵敏性、特异性评估目前尚无统一临床标准。

尽管二代测序技术在微生物检测中有着重要作用，多个指南共识均强调不能取代传统病原学诊断技术，当传统检测手段不能明确致病微生物时，可以开展二代测序作为补充，指导精准治疗，一定程度上能够避免抗菌药物的滥用。本文总结了3例血培养和（或）肝脏组织培养阴性的播散性念珠菌病病例，通过NGS检出念珠菌序列，启动IFD的诊断驱动治疗，取得了良好的治疗效果。但因为病例数较少，需要更多的研究来证实NGS诊断血液肿瘤患者合并CDC的应用价值。相信随着研究的发展，诊断标准趋于完善，质量控制得到提升，成本进一步下降，NGS的应用前景将会更加广阔。
